# Cefazolin Versus Antistaphylococcal Penicillins in Methicillin-Susceptible *Staphylococcus aureus* Bone and Joint Infections: A Narrative Review

**DOI:** 10.3390/antibiotics15090930

**Published:** 2026-09-19

**Authors:** Felix Werneburg, Juliane Beschauner, Laura Isabell Werneburg, Alexander Zeh, Natalia Gutteck, Karl-Stefan Delank

**Affiliations:** 1 Department of Orthopedic and Trauma Surgery, Martin Luther University Halle-Wittenberg, 06108 Halle (Saale), Germany; 2 Department of Prosthodontics and Geriatric Dentistry, Martin Luther University Halle-Wittenberg, 06108 Halle (Saale), Germany

**Keywords:** cefazolin, flucloxacillin, antistaphylococcal penicillins, methicillin-susceptible *Staphylococcus aureus*, bone and joint infection, osteomyelitis, periprosthetic joint infection, inoculum effect, outpatient parenteral antimicrobial therapy, drug safety

## Abstract

Methicillin-susceptible *Staphylococcus aureus* (MSSA) is among the most common pathogens causing bone and joint infections (BJI), which require adequate source control combined with prolonged antimicrobial therapy. For decades, intravenous antistaphylococcal penicillins (ASP) have generally been preferred over cefazolin for MSSA bacteremia, although this practice was based more on convention and historical concerns than on robust comparative evidence. Two recent randomized controlled trials—CloCeBa (2025) and the MSSA domain of the *Staphylococcus aureus* Network Adaptive Platform (SNAP) trial (2026)—have now established that cefazolin is noninferior to ASP for MSSA bacteremia, with an overall more favorable safety profile. Whether these findings extend to BJI remains uncertain: osteoarticular infection was the most common focus in SNAP, yet no focus-specific analyses have been published, and direct comparative evidence in BJI is limited to a few retrospective cohorts. This narrative review summarizes the randomized and observational evidence with emphasis on osteoarticular populations and discusses the cefazolin inoculum effect, bone pharmacokinetics, the safety of prolonged therapy, outpatient practicality and current guidelines. We conclude that cefazolin is a rational first-line option for most patients with MSSA BJI, outline the remaining uncertainties in high-inoculum and implant-associated infection, and propose a focus-specific research agenda.

## 1. Introduction

*Staphylococcus aureus* is one of the most frequently isolated pathogens across the spectrum of bone and joint infections (BJI)—native and vertebral osteomyelitis, septic arthritis, periprosthetic joint infection (PJI) and fracture-related infection—and in most European settings the majority of isolates remain methicillin-susceptible [1,2]. Successful management rests on adequate source control and prolonged antimicrobial therapy, with treatment durations of up to twelve weeks depending on the entity, retained implants and the feasibility of an oral switch [3,4,5,6]. Because therapy extends over weeks, the choice of the intravenous backbone agent affects not only effectiveness but also cumulative toxicity, outpatient deliverability and treatment completion.

For methicillin-susceptible *S. aureus* (MSSA), two β-lactam strategies have coexisted for half a century: the antistaphylococcal penicillins (ASP)—flucloxacillin and cloxacillin in Europe and Australasia, and nafcillin and oxacillin in North America [7]—and the first-generation cephalosporin cefazolin [8]. Both classes are bactericidal, cell-wall-active β-lactams that inhibit penicillin-binding proteins; the ASPs owe their antistaphylococcal activity to bulky acyl side chains—an isoxazolyl ring in oxacillin, cloxacillin and flucloxacillin, a naphthalene ring in nafcillin—that sterically shield the β-lactam ring from staphylococcal penicillinase, whereas cefazolin is hydrolyzed more efficiently by this enzyme at high enzyme and bacterial densities, the biochemical basis of the inoculum effect discussed in Section 4. Cefazolin offers eight-hourly (or continuous-infusion) dosing, a favorable tolerability profile, compatibility with outpatient parenteral antimicrobial therapy (OPAT) and low cost [9,10,11]; historically it was nevertheless often relegated to second-line use, mainly because of concerns about the cefazolin inoculum effect in high-burden infection.

For bacteremia, this equipoise has been resolved. Meta-analyses of the observational literature had already suggested at least equivalent effectiveness and better tolerability of cefazolin [11,12,13,14]; definitive evidence then came from two randomized controlled trials. The French CloCeBa trial demonstrated noninferiority of cefazolin versus cloxacillin with markedly less severe acute kidney injury [15], and the international *Staphylococcus aureus* Network Adaptive Platform (SNAP) trial confirmed noninferiority versus flucloxacillin or cloxacillin for 90-day mortality, again with better renal safety and fewer discontinuations [16,17]. New guideline guidance is expected to follow: at the time of writing, an IDSA/ESCMID guideline on *S. aureus* bacteremia is under development [18].

Bone and joint infections, however, differ from bacteremia in precisely the dimensions that could modify this comparison: treatment courses are several-fold longer; infected bone, sequestra and implant-associated biofilm constitute high-inoculum compartments; success is defined by infection-free survival over months to years rather than 90-day mortality; and much of the therapy can be delivered as OPAT. Direct comparative evidence in BJI is remarkably scarce—to our knowledge, three retrospective cohort studies exist: two on MSSA spinal epidural abscess [19,20] and our own single-center comparison of cefazolin versus flucloxacillin as intravenous monotherapy in mixed MSSA BJI [21]. Although osteoarticular infection was the most common focus in SNAP (32.1% of participants), no focus-specific subgroup analysis has been published [16], and a recent review of the trial results does not directly address bone and joint infections at all [17].

Against this background, we review the evidence on cefazolin versus ASP with a specific focus on BJI: the randomized evidence and its transferability, the direct and indirect comparative data in osteoarticular populations, and the microbiological, pharmacokinetic, safety-related and practical considerations relevant to drug selection, closing with guideline recommendations and a research agenda. We searched PubMed and reference lists of retrieved articles through August 2026 using combinations of drug names, “Staphylococcus aureus” and BJI-related terms, prioritizing randomized trials, comparative cohorts, systematic reviews and guidelines; no formal protocol was registered and no formal risk-of-bias or GRADE assessment was undertaken, in keeping with the narrative format; the limitations that follow from this approach are discussed in Section 10.

## 2. The RCT Era: What SNAP and CloCeBa Answered—And What They Did Not Answer for BJI

### 2.1. The Randomized Trials

The SNAP MSSA domain—part of a pragmatic, investigator-initiated, open-label Bayesian adaptive platform trial conducted at 91 sites in eight countries [22]—randomized 1341 adults with MSSA bacteremia to cefazolin or an isoxazolyl penicillin [16]. Ninety-day mortality was 15.0% with cefazolin versus 17.0% with ASP (adjusted OR 0.81; 95% credible interval 0.59–1.12), meeting noninferiority with a posterior probability of 99.2% and reaching a probability of superiority of 89.8% [16]. The safety results were unambiguous: acute kidney injury (AKI) 13.9% versus 19.6%, serious adverse events 1.8% versus 4.9%, and discontinuation for adverse events 1.6% versus 9.1% [16,17]. The French CloCeBa trial had shown the same pattern: among 315 randomized participants, the composite of therapeutic success at day 90 was met in 75% versus 74% (treatment difference −1%; 95% CI −11 to +9; noninferior), while serious adverse events by the end of study treatment (15% vs. 27%) and acute kidney injury by day 7 (1% vs. 12%) were markedly less frequent with cefazolin [15].

### 2.2. Class-Consistent Nephrotoxicity

In the parallel PSSA domain of SNAP, recruitment was ceased on the recommendation of the data and safety monitoring committee because of increased AKI in participants receiving flucloxacillin or cloxacillin; although the prespecified noninferiority criterion for benzylpenicillin was not formally met, AKI occurred in 11% versus 22% (adjusted OR 0.50; 95% credible interval 0.26–0.94), and the investigators concluded that benzylpenicillin should be preferred [17,23]. Whatever the comparator, prolonged isoxazolyl-penicillin therapy carries a substantial, reproducible renal toxicity burden—an observation of obvious relevance to BJI, where treatment durations are longest.

### 2.3. The Meta-Analytic Backdrop

The trials confirmed what a decade of observational syntheses had suggested. Meta-analyses found equivalent or lower mortality with cefazolin and consistently lower nephrotoxicity, hepatotoxicity and discontinuation rates [11,12,13], and the largest synthesis—30 studies with 15,513 patients, conducted by the SNAP investigators—confirmed lower 30-day mortality (OR 0.73) and markedly lower nephrotoxicity (OR 0.30) consistently across ASP substances [14]. One caveat of the observational era remains influential: in a meta-analysis with trial sequential analysis, relapse was more frequent under cefazolin (OR 1.41; *p* = 0.03) in a population with approximately one-quarter deep-seated infections [13]. Notably, none of these syntheses included a bone-and-joint-specific analysis.

### 2.4. What Remains Unanswered for Bone and Joint Infections

**Figure 1:**
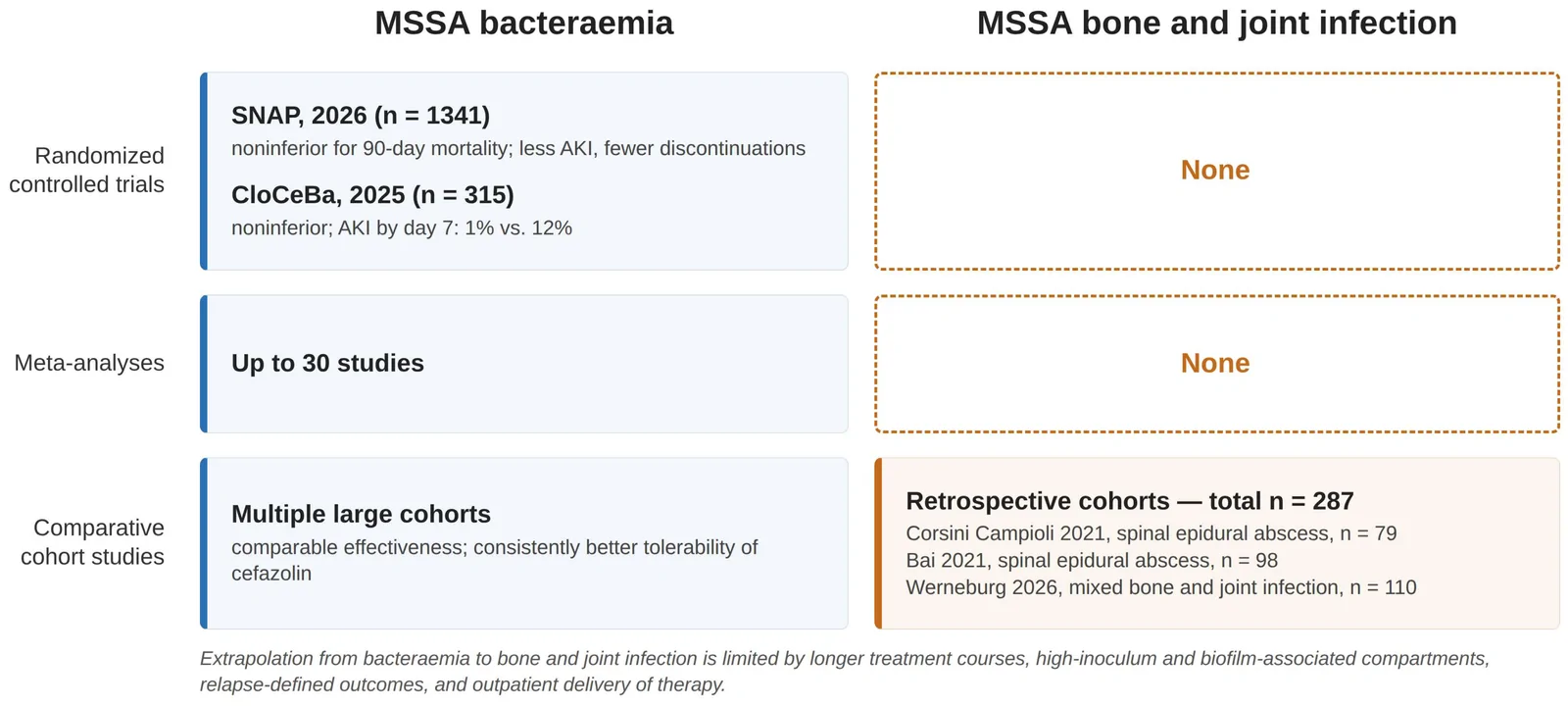
Comparative evidence for cefazolin versus antistaphylococcal penicillins in methicillin-susceptible *Staphylococcus aureus* (MSSA) infections, by level of evidence. In bacteremia (**left**), two randomized controlled trials [15,16] and meta-analyzed observational data [11,12,13,14] have established noninferior effectiveness of cefazolin with superior renal safety and tolerability. In bone and joint infection (**right**), no randomized or meta-analytic evidence exists and direct comparative evidence comprises three retrospective cohort studies with a combined total of 287 patients [19,20,21]. AKI, acute kidney injury.

Five uncertainties remain for the osteoarticular population (Figure 1). First, no focus-specific efficacy data have been published: osteoarticular infection was the most common focus in SNAP (32.1%), yet neither the primary publication nor the accompanying literature reports outcomes for this subgroup [16,17], and in CloCeBa—where arthritis or osteoarthritis constituted the infection focus in 21–28% and deep abscesses in a further 6–11% of participants—focus-specific analyses are equally unavailable [15]. A published osteoarticular analysis would arguably be the most informative next dataset for this field. Second, the trial endpoints are poorly matched to BJI: 90-day mortality is insensitive to relapse, chronicity, implant retention and function over months and years—and the observational relapse signal in deep-seated infection [13] therefore cannot be dismissed on the basis of the RCTs. Third, treatment durations differ fundamentally, and cumulative-exposure toxicities scale with duration. Fourth, the microbiological concern is concentrated in BJI-type compartments: the inoculum effect is a phenomenon of high bacterial density and thus of greatest theoretical relevance in bone infections, abscesses and biofilm-associated infections. Fifth, implant- and biofilm-associated infections are not separately represented in the trials, and adjunctive strategies typical of BJI management (rifampicin combinations, staged revision, suppression) were outside their scope. The last point deserves particular emphasis: bacteria within biofilm are phenotypically tolerant to cell-wall-active agents largely irrespective of β-lactam class, so the choice between cefazolin and an ASP concerns the systemic backbone directed against planktonic and tissue-phase organisms, while biofilm control remains primarily surgical and combination-based.

## 3. Direct Comparative Evidence in Bone and Joint Infections

### 3.1. Spinal Epidural Abscess: Two Comparative Cohorts

Two independent 2021 cohorts concern the same high-stakes syndrome. In a retrospective multicentre cohort of 79 adults with MSSA spinal epidural abscesses (ASP *n* = 34, cefazolin *n* = 45), there were no significant differences in treatment failure at weeks 6 and 12, 30-day mortality (5.9% vs. 2%) or 90-day recurrence (9.4% vs. 11.4%; ASP vs. cefazolin throughout) [19]. A Canadian cohort comparing cefazolin (*n* = 50) with cloxacillin (*n* = 48) was likewise reassuring—90-day mortality 8% versus 13%, failure 12% versus 19%, recurrence 2% versus 8% (all nonsignificant), serious adverse reactions only under cloxacillin—and concluded that cefazolin “may be considered as a first-line treatment” [20]. The fact that both cohorts concern an anatomical compartment in which concerns about cefazolin’s central nervous system penetration might have been expected to manifest lends their concordant results additional weight (Section 5.4).

### 3.2. The Halle Cohort: Cefazolin Versus Flucloxacillin as Intravenous Monotherapy in Mixed BJI

Our own retrospective single-center study compared cefazolin (*n* = 64) and flucloxacillin (*n* = 46) as targeted intravenous inpatient monotherapy in 110 adults with culture-confirmed, monomicrobial MSSA BJI—a deliberately selected population excluding rifampicin-based combinations, OPAT, polymicrobial infection and concurrent bacteremia or endocarditis; all endpoints were a priori exploratory [21]. No statistically significant differences in effectiveness were detected: 30-day clinical success (alive and infection-free) 89.1% versus 80.4% (absolute risk difference [ARD] +8.6 percentage points; 95% CI −4.7 to +23.3) and one-year clinical success 79.7% versus 65.2% (ARD +14.5; 95% CI −2.2 to +31.0) [21]. Peri-treatment AKI occurred in 19.0% versus 30.4% (adjusted OR for flucloxacillin 2.27; 95% CI 0.87–5.91)—despite more frequent nephrotoxic co-medication in the cefazolin group—with the excess confined to stage 1 injury. Point estimates favored cefazolin throughout, but confidence intervals were wide, and results were directionally consistent across implant-associated and native infections. Together with the spinal epidural abscess cohorts, this study constitutes—to our knowledge—the entirety of published direct comparative evidence in osteoarticular populations: concordant with the randomized bacteremia data, but hypothesis-generating rather than confirmatory.

### 3.3. Osteoarticular Signals Within the Bacteremia Cohorts

Several classical bacteremia cohorts carry indirect information. The cohort with the highest osteoarticular share (41% of infection sources; *n* = 93) found clinical cure in 95% (cefazolin) versus 88% (oxacillin; *p* = 0.25), with better tolerability of cefazolin [24], and an analysis restricted to deep-seated MSSA bloodstream infection (18% bone/joint) found no efficacy disadvantage of cefazolin precisely in the high-inoculum situation (failure 15.6% vs. 20.0%) [25]. Conversely, the Canadian cohort of Bai et al. reported that all six relapses occurred in cefazolin-treated patients with deep-seated infection—the most frequently cited relapse signal in this literature [26]. The largest cohorts point in the same direction as the trials: there was lower mortality with cefazolin in a US Veterans Affairs cohort of 3167 patients (osteomyelitis in 12–13%) [27], and equivalent mortality in an Australia/New Zealand cohort of 7312 episodes comparing the same drug pair as our study—the analysis whose authors called for the trial that became SNAP [28]. Finally, in the OPAT setting, premature discontinuation was five times more frequent under nafcillin than cefazolin (33.8% vs. 6.7%) [10]—directly relevant to completing multi-week osteoarticular courses. Viewed quantitatively, roughly 500 randomized participants with an osteoarticular focus are embedded–so far unanalyzed–within SNAP and CloCeBa alone (approximately 430 in SNAP [16] and 60–80 with arthritis or osteoarthritis in CloCeBa [15]), alongside the osteoarticular fractions (12–41%) of the major observational cohorts [24,25,26,27,28] (Table 1): a substantial reservoir of existing but untapped comparative information.

### 3.4. Pediatric Osteoarticular Infection

Pediatric guidelines have long listed first-generation cephalosporins and ASP as co-equal first-line options with early oral transition [29,30]. Direct comparative evidence is lacking in children as well. Notably, in a surveillance study of 250 children with MSSA osteoarticular infection, a cefazolin inoculum effect (14.4% of isolates) was associated with progression to chronic osteomyelitis irrespective of the definitive antibiotic chosen—arguing for strain virulence rather than cefazolin failure as the driver [31].

### 3.5. Infective Endocarditis as a High-Inoculum Analog

Because vegetations share the high-inoculum biology invoked against cefazolin, endocarditis serves as a stress test for extrapolation to bone. A 2025 systematic review of seven observational cohorts found lower 30-day mortality with cefazolin (OR 0.49) and comparable relapse rates [32], whereas a French three-center case series of 216 MSSA endocarditis episodes found no overall outcome difference between the two classes, but reported first-month mortality of 40.3% versus 19.4% depending on the presence of an inoculum effect against the β-lactam actually received (adjusted HR 2.84; 95% CI 1.28–6.30) [33]—mirroring the osteoarticular question in miniature: aggregate outcomes were reassuring, but a biologically plausible residual concern remained in the highest-inoculum niches (Section 4).

### 3.6. Strength and Applicability of the Evidence by BJI Category

The comparative evidence base differs markedly across the clinical entities grouped under BJI, and Table 2 summarizes its strength and applicability by category. For no single entity does the direct evidence rise above retrospective cohort data; certainty is therefore judged narratively, informed by the GRADE domains of study design, directness and precision, rather than by formal rating. The pattern is nevertheless consistent: spinal epidural abscess and mixed adult BJI carry the only dedicated comparative data [19,20,21]; every other category relies on extrapolation—from the bacteremia trials [15,16], from the osteoarticular fractions of the observational cohorts [24,25,26,27,28], or from guidelines that predate the randomized era—and in none is there a comparative signal that contradicts the bacteremia findings.

## 4. Microbiological Considerations: The Inoculum Effect and Susceptibility Testing

### 4.1. Definition, Mechanism and Prevalence

The principal microbiological argument against cefazolin is the cefazolin inoculum effect (CzIE): a rise in the minimum inhibitory concentration to ≥16 µg/mL when testing is performed at high bacterial density (~5 × 10^7^ CFU/mL) rather than at the standard inoculum, reflecting accelerated hydrolysis by staphylococcal β-lactamase—most efficiently by the type A enzyme—at high bacterial burden. In the founding systematic characterization, 19% of clinical MSSA isolates displayed a pronounced CzIE, with the cefazolin MIC90 rising from 2 to 32 µg/mL at high inoculum [34]; in an experimental endocarditis model with a CzIE-positive strain, cefazolin was subsequently shown to be inferior to nafcillin, providing in vivo plausibility for concern in high-inoculum niches [35]. The effect is genotype-dependent—observed in approximately 78% of blaZ type A-, 53% of type C-, but only 10% of type B-carrying isolates [36]—and its prevalence varies widely with region and setting—19% in the founding characterization [34] and 14.4% in a pediatric osteoarticular series [31], with the highest rates reported in Latin America [37].

### 4.2. Clinical Relevance: An Unresolved Debate with Particular Weight in BJI

Whether the CzIE worsens outcomes remains contested. A Latin American bacteremia cohort reported an adjusted 2.65-fold increase in 30-day mortality with CzIE-positive isolates [38], whereas a 2024 systematic review of 23 studies found no consistent mortality association and recommended against routine testing [39]. The newest data move the controversy toward the osteoarticular field: in a multicentre cohort of 259 cefazolin-treated patients with serious MSSA infection based on blood or deep sterile-site cultures, the CzIE (prevalence 35.5%) was not associated with 90-day mortality (20.7% vs. 22.2%) but it was independently associated with microbiological treatment failure (20.7% vs. 6.0%; adjusted sub-distribution HR 3.12; 95% CI 1.38–7.08) [40]. Randomized data now feed directly into this debate: in CloCeBa, the noninferiority of cefazolin was not demonstrated in the (non-prespecified) subgroup infected with blaZ type A-carrying strains (therapeutic success 19/29 vs. 21/27; difference −12%; 95% CI −34 to +12), although early microbiological failure did not differ significantly by blaZ type A carriage and noninferiority was met in all other participants [15]. In endocarditis, the French inoculum-effect mortality signal [33] is countered by an analysis arguing that clinical characteristics and medical–surgical management outweigh the inoculum effect and genotype [41]; and in pediatric osteoarticular infection, the CzIE predicted chronicity irrespective of drug choice [31]. For BJI, three points deserve emphasis: the biology of the CzIE is maximally relevant to sequestra, abscesses and biofilm, adequate surgical source control appears to attenuate its clinical expression [39,41], and no comparative osteoarticular outcome data stratified by CzIE status exist—the decisive question is, strictly speaking, unanswered. The prevalence heterogeneity itself carries interpretive weight: with reported rates spanning roughly 14% to more than 35% between settings and assays [31,34,37,40], aggregate results from trials conducted predominantly in lower-prevalence regions may not capture CzIE-associated risk where type A β-lactamase carriage is common, and single-region cohort findings—whether reassuring or alarming—generalize poorly in either direction [36,37].

### 4.3. Susceptibility Testing and the Case for Targeted CzIE Testing

In routine microbiology, β-lactam susceptibility of MSSA is inferred from cefoxitin (or oxacillin) testing; cefazolin is not tested individually [42], and this inference remains sound—cefazolin susceptibility is essentially universal, without MIC creep [43]. Standard testing cannot, by design, detect the CzIE; a rapid (~3 h) colorimetric β-lactamase assay has been developed for this purpose and validated on 689 bloodstream isolates (overall sensitivity 82.5% and specificity 88.9%, rising to 92.7% sensitivity for type A β-lactamase carriers) [44]. Routine CzIE testing is currently neither standard practice nor guideline-endorsed [39], and we do not advocate it for unselected BJI. A rational niche can be argued for persistent or relapsing osteoarticular infection under cefazolin despite adequate source control, where either targeted testing or an empirical class switch to an ASP appears reasonable pending better data.

## 5. Pharmacokinetics, Bone Penetration and Mode of Infusion

### 5.1. Pharmacodynamic Targets and Free Drug

β-Lactam efficacy is driven by the time the free drug concentration exceeds the MIC (fT > MIC); for deep-seated infection, 100% fT > MIC is increasingly advocated as a minimum target, although the optimal PK/PD target remains undefined (Section 5.3). The classes differ in protein binding—approximately 75–85% for cefazolin versus 93–96% for isoxazolyl penicillins—and empirical data have settled the practical implication: in a prospective head-to-head cohort of invasive MSSA infections, the pharmacodynamic target (free concentration ≥2 × MIC for ≥50% of the interval) was missed by 26% of flucloxacillin- but only 4% of cefazolin-treated patients (*p* = 0.002), with median free concentrations of 2.6 versus 15.4 mg/L [45].

### 5.2. Bone and Joint Penetration

The most comprehensive review of target-site data classifies both cefazolin and the isoxazolyl penicillins among agents with adequate bone penetration, but its pooled figures describe an asymmetry: average cancellous bone and joint concentrations of 75.4 and 112.2 µg/mL for cefazolin, versus cortical bone concentrations of ~4 µg/mL for oxacillin (synovial fluid 3.4 µg/mL), ~3.8 µg/mL in bone for cloxacillin and dicloxacillin (joint 0.5 µg/mL), and 89.5 µg/mL in cancellous bone for flucloxacillin after repeated 2 g dosing, whose joint-space penetration was nevertheless flagged as poor, albeit based on a small low-dose study; for nafcillin, no human bone data were identified [46]. These heterogeneous perioperative values are not directly comparable; their common denominator is that all agents with available data exceed the MIC90 of susceptible staphylococci in bone, with the review cautioning that the high protein binding of the isoxazolyl penicillins may limit the active free fraction at the target site [46]. The most informative single dataset for cefazolin stems from the continuous-infusion therapy of BJI: at a median dose of 6 g/day, median serum and bone concentrations of 63 µg/mL and 13.5 µg/g (bone-to-serum ratio 0.25) were achieved alongside an ~81% clinical success rate [9]. For flucloxacillin, conversely, a population pharmacokinetic model—built on microdialysis measurements of free bone and soft-tissue concentrations in a porcine model bridged to human plasma pharmacokinetics, rather than on direct target-site measurements in patients with BJI—concluded that standard intermittent dosing of 4 g/day does not reliably achieve therapeutic free target-site concentrations, whereas continuous infusion or escalation to 8–12 g/day restores predicted target attainment [47].

### 5.3. Continuous Infusion and Practical Dosing

Both classes are amenable to continuous infusion. For cloxacillin in MSSA BJI, continuous infusion achieved 100% fT > MIC whereas six-hourly intermittent dosing was suboptimal at equal daily doses [48]; for cefazolin, a recent population pharmacokinetic study reported complete target attainment in 86% of patients under standard dosing, identified renal function as the dominant covariate, and advocated for model-informed dosing [49]. These study-level findings align with the recent multidisciplinary consensus guidance on β-lactam dose individualization in acutely ill patients, which recommends a minimum target of 100% fT > MIC, while emphasizing that optimal PK/PD targets remain to be defined; the consensus guidance also supports individualized dosing and therapeutic drug monitoring where pharmacokinetic variability is high [50]. In practice, cefazolin attains its targets with conventional every-eight-hour dosing in most patients, whereas the short half-life and high protein binding of the isoxazolyl penicillins push regimens toward four- to six-hourly administration, high daily doses or continuous infusion [45,47,48]—an asymmetry with direct logistic consequences for prolonged and outpatient therapy (Section 7).

### 5.4. The Central Nervous System Question

The traditional argument for preferring an ASP in infections located near the neuraxis has been cefazolin’s reputedly poor cerebrospinal fluid penetration. Newer data qualify this only in part: in a prospective pharmacokinetic evaluation of 15 neurocritical-care patients with external ventricular drains—who received standard-dose cefazolin as prophylaxis, not as treatment of established central nervous system infection—cerebrospinal fluid concentrations were detectable but variable, and the authors judged cefazolin as potentially viable for susceptible central nervous system infections [51]; these are pharmacokinetic rather than efficacy data. What is more directly relevant to BJI, the two spinal epidural abscess cohorts found no efficacy disadvantage of cefazolin in a compartment adjacent to—though distinct from—the meninges [19,20]. High-dose cefazolin is therefore defensible in neuraxis-adjacent BJI such as spinal epidural abscesses, whereas for meningitis or extensive intradural diseases—entities beyond the scope of BJI—comparative outcome data are lacking and many clinicians reasonably continue to prefer an ASP.

## 6. Safety of Prolonged Therapy

Because BJI requires weeks of high-dose therapy, cumulative and idiosyncratic toxicities weigh heavily—and treatment completion itself becomes an outcome: in the OPAT setting, discontinuation was five times more frequent under nafcillin than cefazolin [10], and discontinuation for adverse events consistently favored cefazolin in the trials [15,16]. The principal safety considerations, drug-specific toxicities, and practical monitoring requirements of cefazolin and ASPs during intravenous therapy are summarized in Table 3.

### 6.1. Hepatotoxicity

Flucloxacillin is a paradigmatic cause of idiosyncratic, typically cholestatic drug-induced liver injury (DILI); its association with HLA-B*57:01—an approximately 80-fold risk increase—is among the strongest known pharmacogenetic associations [52], and nationwide cohort data confirm a sevenfold increased risk concentrated in the first 45 days of exposure (HR 7.32; 95% CI 4.1–13.0), corresponding to roughly 11 additional cases per 100,000 treatment courses [53]. Population-based analyses quantify the absolute risk at roughly 8.5 laboratory-confirmed cases per 100,000 users and identify higher age (relative risk ≈ 23 beyond 70 years) and repeated, consecutive prescriptions (relative risk ≈ 9) as the dominant risk factors, with cholestatic injury typically manifesting only after several weeks of exposure [54]—a profile of obvious concern for four- to twelve-week BJI courses. Among the American ASPs, hepatotoxicity is more frequent under oxacillin [55]. Clinically apparent hepatotoxicity under cefazolin is rare; the similar rates of laboratory-defined liver injury in SNAP [16] do not contradict the DILI signal, which concerns rarer, partly delayed idiosyncratic injury that short bacteremia courses are ill-suited to capture.

### 6.2. Nephrotoxicity and Acute Interstitial Nephritis

The renal gradient is the most consistently documented safety finding: meta-analytically, nephrotoxicity was substantially less frequent under cefazolin [11]; in a dedicated cohort, AKI occurred in 32% of cases under nafcillin versus 13% of cases under cefazolin (adjusted OR 2.74), with markedly higher costs [56]; the randomized trials removed residual doubt (AKI 13.9% vs. 19.6% in SNAP [16]; AKI by day 7 in 1% vs. 12% in CloCeBa [15]; 11% vs. 22% in the PSSA domain [23]). Our BJI cohort is directionally consistent [21]. ASP nephrotoxicity is classically attributed to acute interstitial nephritis, although AKI events in the randomized trials were not systematically biopsy-characterized, so the relative contributions of interstitial nephritis and other mechanisms remain presumptive, and it can supervene surprisingly early: in a biopsy-proven series—in which flucloxacillin was the most frequent culprit and MSSA osteomyelitis figured among the index indications—the median time from exposure to AKI was only 2.5 days (IQR 2–8) [57]. Creatinine monitoring from the outset of therapy and at least weekly thereafter is therefore prudent under either agent, with a low threshold for re-evaluation under an ASP.

### 6.3. Cytopenias, Electrolytes, Local Tolerability and Neurotoxicity

Neutropenia is a class effect of prolonged high-dose β-lactam therapy, typically after more than two weeks [58]; weekly blood counts are standard under either agent. Hypokalemia is a frequent and underappreciated complication of high-dose intravenous flucloxacillin—occurring in 42% of flucloxacillin-treated patients versus 14% under the comparator ceftriaxone (comparative data against cefazolin are lacking), typically within the first five days of therapy and more pronounced in women [59]—and the sodium load of high-dose regimens deserves attention in cardiac and renal comorbidity. Nafcillin is associated with infusion-site phlebitis and documented tissue necrosis after extravasation [60]. For cefazolin, the principal dose-dependent toxicity is neurotoxicity upon accumulation in renal impairment, mandating consistent renal dose adjustment [61].

### 6.4. Hypersensitivity and Drug Interactions

Cefazolin’s R1 side chain is shared with neither penicillins nor other cephalosporins, and R1 side-chain patterns explain most clinically observed cross-reactivity, which for cefazolin is rare though not absent [62]; in meta-analysis, dual allergy to both a penicillin and cefazolin is documented in approximately 0.7% of patients, and is substantially more frequent (approximately 10%) among patients with skin-test-confirmed cefazolin allergies [62]; perioperative data support the safety of cefazolin in most patients carrying an unverified penicillin allergy label [63]—a tangible advantage for roughly one in ten patients who carry such a label, and in whom ASPs are contraindicated if the allergy is genuine. Conversely, flucloxacillin is a clinically relevant inducer of drug metabolism via the pregnane X receptor, reducing voriconazole exposure to subtherapeutic levels—a combination explicitly to be avoided [64]; no comparable induction is known for cefazolin.

**Table 3:** Practical safety and monitoring profile of cefazolin versus antistaphylococcal penicillins under prolonged intravenous therapy. AIN, acute interstitial nephritis; AKI, acute kidney injury; CI, continuous infusion; DILI, drug-induced liver injury.

Domain	Cefazolin	Flucloxacillin/Cloxacillin	Oxacillin/Nafcillin
Standard i.v. dosing	2 g q8h (or ~6 g/day CI); renal adjustment mandatory [9,49]	1–2 g q4–6h or 8–12 g/day CI [47,48]	2 g q4–6h
AKI/AIN [11,15,16,56,57]	Low; reference class	Consistently higher (AIN; RCT-confirmed)	Consistently higher
DILI [52,53,54,55]	Rare	Idiosyncratic cholestatic DILI; HLA-B*57:01-associated; risk increased with higher age and repeated courses	Transaminitis frequent (oxacillin > nafcillin)
Hypokalemia/sodium load [59] ^a^	Uncommon	Frequent under high-dose i.v. therapy ^a^	Reported (limited data)
Neutropenia (>2 weeks) [58]	Yes; monitor	Yes; monitor	Yes; monitor
Phlebitis/extravasation [60]	Low	Moderate	High (nafcillin: necrosis risk)
Neurotoxicity [61]	Seizures upon renal accumulation; dose adjustment required	Low	Low
Penicillin allergy label [62,63]	Generally usable (unique R1 side chain)	Contraindicated in true penicillin allergy	Contraindicated in true penicillin allergy
Key interactions [64]	None comparable	PXR induction: voriconazole/azole failure	Limited data
Monitoring suggestion	Creatinine + blood count weekly	Creatinine, potassium, liver panel + blood count weekly	Creatinine, liver panel + blood count weekly

^a^ Comparative frequency data for hypokalemia derive from a cohort with ceftriaxone, not cefazolin, as the comparator [59].

## 7. Practical Considerations: Outpatient Therapy, Logistics and Stewardship

After the initial inpatient phase, much of BJI therapy is delivered as OPAT, making stability, dosing frequency and infusion logistics co-determinants of drug choice. Stability in elastomeric devices limits the ambulatory use of several β-lactams, and the supporting evidence base is heterogeneous [65]. Cefazolin-based OPAT programs report success rates around 90% [66]; for flucloxacillin, a citrate-buffered formulation retaining >95% potency over 14 days (including 24 h at body-worn temperature) meets the UK Yellow Cover Document standard and has enabled continuous-infusion OPAT [67,68], for which a UK service evaluation—including osteomyelitis and septic arthritis—reported treatment-goal attainment in 95% of patients [69]. The tolerability asymmetry documented under OPAT conditions [10] carries particular weight here: every discontinuation in week four of a planned six-week course forces a class switch, a readmission or a compromise on treatment adequacy. Dosing logistics follow the pharmacokinetics (Section 5.3): three short infusions or one elastomeric device per day for cefazolin, versus four- to six-hourly administration or high-volume continuous infusion for the isoxazolyl penicillins, with correspondingly higher nursing workload—and higher per-case costs for the ASP in comparative analyses [56].

Two systemic aspects complete the picture. Supply security cuts both ways—shortages have affected cefazolin as well as the regionally fragmented ASPs [7,70]—so institutional pathways should specify an explicit second-line agent of the respective other class. From a stewardship perspective, both classes are WHO AWaRe Access agents, and the WHO essential-medicines expert committee lists cloxacillin as the first-choice and cefazolin among the second-choice agents for bone and joint infections [71]—a hierarchy that predates the randomized era—while the incremental ecological benefit of the ASPs over cefazolin, itself a narrow-spectrum Access agent, remains theoretical. The few comparative ecological data available support this reading: in CloCeBa, a *Clostridioides difficile* infection occurred in 2% of participants in both arms [15]; comparative data on broader microbiome effects and on resistance selection under prolonged therapy are lacking, as are dedicated cost-effectiveness analyses in BJI; existing economic signals (higher per-case costs and nursing workload under ASPs) derive from bacteremia and OPAT settings [10,56].

## 8. Current Guideline Recommendations

Across current guidance, cefazolin and the ASPs are listed as co-equal first-line options for MSSA osteoarticular infections; without operationalizing the safety gradient documented above, the IDSA guidelines on prosthetic joint infection and native vertebral osteomyelitis name both classes side by side [3,4], the German PEG S2k recommendation treats flucloxacillin and cefazolin as equivalent for staphylococcal BJI [72], and the 2026 AWMF guideline on fracture-related infection specifies durations but no MSSA agent preference—an explicit gap [73]. Pediatric guidance is likewise co-equal, with emphasis on early oral transition [29,30]. Looking ahead, a first joint IDSA/ESCMID guideline on *S. aureus* bacteremia was under development at the time of writing (last verified 8 September 2026) [18]; it can be anticipated to be the first major guidance document to weigh the randomized evidence, and its publication will provide a natural occasion to reappraise the co-equal positioning of the two classes in osteoarticular recommendations as well.

Two contextual trends sharpen rather than blunt the comparison. The intravenous phase itself is shrinking: OVIVA established the noninferiority of early oral therapy in BJI [6], SABATO that of an early oral switch in low-risk *S. aureus* bloodstream infection [74], and the trial by Bernard et al. the adequacy of six-week regimens in vertebral osteomyelitis [5]—three distinct questions whose combined effect is to compress the intravenous phase—and the SNAP early-oral-switch domain will refine this further for staphylococcal disease [75]. Paradoxically, this concentrates the decision: when intravenous therapy is compressed into the first one to six weeks, tolerability, renal safety and OPAT deliverability during precisely that window dominate the clinical calculus. The co-equal listing should therefore be read in its historical context: it largely reflects the observational evidence base that preceded the randomized trials, and updated guidance will need to weigh the new data explicitly.

## 9. Open Questions and Future Directions

Four research priorities emerge. First—and most immediately actionable—a focus-specific analysis of the SNAP osteoarticular subgroup, which makes up nearly one-third of a 1341-patient randomized cohort, would constitute by far the largest body of randomized comparative data in osteoarticular MSSA infection; its publication should be a priority for the field [16]. Because SNAP was designed and powered for bacteremia, such an analysis should be framed as a transportability exercise rather than a definitive subgroup verdict—ideally with prespecified osteoarticular definitions, reporting of focus-specific baseline characteristics and event counts, assessment of treatment-by-focus interaction, and standardization or overlap weighting toward a BJI-relevant covariate distribution. Second, BJI-specific comparative research with endpoints that match the disease such as infection-free survival at one to two years, implant retention, reoperation and function, is needed, whether it is done using registries, target-trial emulation or a dedicated osteoarticular domain within an adaptive platform; small comparative cohorts such as the spinal epidural abscess studies and our own should be understood as building blocks for pooled analyses rather than stand-alone evidence [19,20,21]. Third, the clinical role of CzIE testing in BJI remains to be defined: rapid assays exist [44], and persistent or relapsing infection under cefazolin despite source control is the setting in which stratified studies—ideally with blaZ genotyping—would be most informative [36,40]. Fourth, pharmacokinetically guided delivery of both classes (continuous infusion, therapeutic drug monitoring, model-informed dosing) deserves systematic evaluation in the multi-week BJI setting, where target attainment, cumulative toxicity and outpatient logistics intersect [45,47,48,49]. Adjunctive and biofilm-directed strategies—from local antimicrobial delivery to physical anti-biofilm approaches—form a rapidly evolving field of their own but lie outside the scope of the systemic-backbone question addressed here.

## 10. Limitations of This Review

This review has several limitations. First, it is narrative by design: no protocol was registered, and no formal risk-of-bias assessment or GRADE rating was performed. Although the search strategy is described transparently (Section 1) and all quantitative statements were verified against the primary publications, the selection and weighting of evidence remain subject to judgment—including the potential for interpretation bias arising from our authorship of one of the three comparative BJI cohorts [21]. Second, the central inference is indirect: the randomized evidence stems from bacteremia trials whose endpoints, treatment durations and populations differ systematically from BJI (Section 2.4), and the direct BJI evidence comprises three retrospective cohorts with a combined 287 patients, wide confidence intervals and a susceptibility to confounding by indication and selection [19,20,21]. Third, the prevalence and clinical expression of the cefazolin inoculum effect vary widely between regions and assays (Section 4), limiting the transferability of any single cohort’s findings—the reassuring ones included. Fourth, comparative data on ecological outcomes, microbiome effects and cost-effectiveness in BJI are essentially absent (Section 7), and regional differences in drug availability constrain the generalizability of any first-line recommendation. These limitations are themselves an argument for the focus-specific research agenda outlined above.

## 11. Conclusions

For MSSA bacteremia, the randomized evidence has effectively settled the comparative question for the agents studied: cefazolin is noninferior to cloxacillin and flucloxacillin, and consistently safer with respect to renal injury and treatment discontinuation; for nafcillin and oxacillin, randomized comparisons are lacking, although the observational evidence points in the same direction [11,12,13,14,27]. For bone and joint infections, direct comparative evidence remains limited to three retrospective cohorts, all directionally concordant with the randomized data [19,20,21]. This indirect but coherent body of evidence—together with cefazolin’s reliable pharmacokinetic target attainment, adequate bone concentrations, favorable safety profile under prolonged therapy, usability in penicillin-allergic patients and outpatient practicality—supports cefazolin as a rational first-line option for most patients with MSSA BJI. Residual uncertainty is concentrated where the bacterial inoculum is highest and surgical source control incomplete; there, and where a cefazolin inoculum effect is documented or suspected, the antistaphylococcal penicillins remain fully appropriate alternatives. Pending focus-specific evidence—foremost an osteoarticular analysis from SNAP—the choice should be individualized on renal risk, hepatic risk factors, allergy status, infusion logistics and local availability, with either agent dosed adequately and monitored throughout the treatment course.

## Figures and Tables

**Table 1:** Comparative clinical studies of cefazolin versus antistaphylococcal penicillins with relevance to bone and joint infection. ARD, absolute risk difference; AKI, acute kidney injury; ASP, antistaphylococcal penicillin(s); CFZ, cefazolin; n.r., not reported; n.s., not significant; SEA, spinal epidural abscess.

Study [Ref]	Design/Population	n (CFZ/ASP)	Osteoarticular Share	Key Findings	Comment
SNAP MSSA domain, 2026 [16]	Platform RCT, MSSA bacteremia, 91 sites	671/670	32.1% (most common focus)	90-day mortality 15.0% vs. 17.0% (noninferior, aOR 0.81); AKI 13.9% vs. 19.6%; discontinuation 1.6% vs. 9.1%	No focus-specific subgroup published
CloCeBa, 2025 [15]	Multicentre RCT, MSSA bacteremia (France, 21 sites)	146/146 analyzed (315 randomized)	Arthritis/osteoarthritis 21–28%; deep abscess 6–11%	Day-90 success 75% vs. 74% (noninferior); AKI by day 7 1% vs. 12%; serious AEs 15% vs. 27%	No focus-specific outcome analyses; noninferiority not shown in blaZ type A subgroup
Corsini Campioli et al., 2021 [19]	Retrospective multicentre (Mayo), MSSA SEA	45/34	100% (SEA)	No differences in failure (weeks 6/12), mortality, 90-day recurrence	Focus-specific comparative cohort
Bai et al., 2021 [20]	Retrospective cohort (Ontario), MSSA SEA	50/48 (cloxacillin)	100% (SEA)	90-day mortality 8% vs. 13%; failure 12% vs. 19%; recurrence 2% vs. 8% (all n.s.); serious ADR 0% vs. 4%	Authors: cefazolin ‘may be considered as a first-line treatment’
Werneburg et al., 2026 [21]	Retrospective single-center, MSSA BJI, i.v. inpatient monotherapy	64/46	100%	1-year clinical success 79.7% vs. 65.2% (ARD +14.5, 95% CI −2.2 to +31.0; n.s.); AKI 19.0% vs. 30.4% (adjusted OR 2.27, 0.87–5.91)	Selected monotherapy cohort; exploratory endpoints
Li et al., 2014 [24]	Retrospective 2-center, complicated MSSA bacteremia	59/34	41% of sources	Cure 95% vs. 88% (n.s.); better tolerability of CFZ	Highest osteoarticular share of bacteremia cohorts
Rao et al., 2015 [25]	Retrospective 2-center, deep-seated MSSA BSI	103/58	18% bone/joint	Failure in deep-seated infection 15.6% vs. 20.0% (n.s.)	No CFZ disadvantage in high-inoculum setting
Bai et al., 2015 [26]	Retrospective multicentre (Canada)	105/249	n.r. (deep-seated subset)	90-day mortality aHR 0.58 (n.s.); all 6 relapses under CFZ in deep-seated infection	Most-cited relapse signal
McDanel et al., 2017 [27]	Retrospective, 119 VA hospitals	1163/2004	Osteomyelitis 12–13%	30-day mortality HR 0.63; 90-day HR 0.77 (favoring CFZ)	No osteomyelitis-specific effect estimate
Davis et al., 2018 [28]	Retrospective laboratory-based (AUS/NZ)	792/6520	n.r.	30-day mortality 10.7% vs. 11.2% (OR 0.93)	Same drug pair as [21]; triggered SNAP

**Table 2:** Strength and applicability of the comparative evidence for cefazolin versus antistaphylococcal penicillins, by category of bone and joint infection. Certainty is judged narratively, informed by the GRADE domains of study design, directness and precision; no formal GRADE rating was performed. AHO, acute hematogenous osteomyelitis; ASP, antistaphylococcal penicillin(s); BJI, bone and joint infection; CzIE, cefazolin inoculum effect; FRI, fracture-related infection; MSSA, methicillin-susceptible *Staphylococcus aureus*; PJI, periprosthetic joint infection; RCT, randomized controlled trial; SEA, spinal epidural abscess.

BJI Category	Direct Comparative Evidence	Indirect and Supporting Evidence	Certainty of Comparative Evidence	Practical Implication
Native septic arthritis	None dedicated; native subset of the mixed BJI cohort [21]	Arthritis/osteoarthritis focus in 21–28% of CloCeBa participants, unanalyzed [15]; bacteremia cohorts [24,25]	Very low (retrospective subsets; indirect randomized evidence)	Either agent per current guidance; extrapolation from bacteremia favors cefazolin on safety
Vertebral osteomyelitis and SEA	Two SEA cohorts, *n* = 177 [19,20]; subset of the mixed BJI cohort [21]	Deep-seated bacteremia analyses [25,26]; neuraxis-adjacent pharmacokinetics (Section 5.4)	Low to very low (consistent, but retrospective and imprecise)	Most directly supported BJI category; concordant reassurance for cefazolin
Native long-bone and chronic osteomyelitis	Subsets of the comparative cohorts [21,24,25,27]	Osteomyelitis share 12–13% in the largest cohort [27]; CzIE chronicity signal (Section 4)	Very low (no entity-specific analyses)	Agent choice on safety and logistics; source control paramount
PJI	None dedicated; implant-associated subset of the mixed BJI cohort [21]	Biofilm tolerance to both classes (Section 2.4); co-equal guideline listing [4]	Very low (systemic backbone only; biofilm biology outside RCT scope)	Backbone choice per safety profile; surgical and combination strategy unchanged [2,4]
FRI	None dedicated; subset of the mixed BJI cohort [21]	Guidance specifies durations but no MSSA agent preference (Section 8)	Very low	Explicit evidence gap; institutional pathways advisable
Pediatric AHO	None	Co-equal guidelines with early oral switch [29,30]; CzIE predicted chronicity irrespective of agent [31]	Very low	Either agent; early oral transition dominates the decision

## Data Availability

No new data were created or analyzed in this study. Data sharing is not applicable to this article.
